# Nailfold videocapillaroscopy micro-haemorrhage and giant capillary counting as an accurate approach for a steady state definition of disease activity in systemic sclerosis

**DOI:** 10.1186/s13075-014-0462-8

**Published:** 2014-10-09

**Authors:** Domenico Sambataro, Gianluca Sambataro, Eleonora Zaccara, Wanda Maglione, Riccardo Polosa, Antonella MV Afeltra, Claudio Vitali, Nicoletta Del Papa

**Affiliations:** U.O.C. Day Hospital Reumatologia, Ospedale Gaetano Pini, Via Gaetano Pini, 9-20122 Milano, Italy; Policlinico Universitario di Catania, Via Santa Sofia, 78, 95123 Catania, Italy; Policlinico Universitario Campus Bio-Medico di Roma, Via Álvaro del Portillo, 200, 00144 Rome, Italy; Istituto San Giuseppe, Via Alla Fonte, 7, 23900 Lecco, Italy

## Abstract

**Introduction:**

Nailfold videocapillaroscopy (NVC) in systemic sclerosis (SSc) is a procedure commonly used for patient classification and subsetting, but not to define disease activity (DA). This study aimed to evaluate whether the number of micro-haemorrhages (MHE), micro-thrombosis (MT), giant capillaries (GC), and normal/dilated capillaries (Cs) in NVC could predict DA in SSc.

**Methods:**

Eight-finger NVC was performed in 107 patients with SSc, and the total number of MHE/MT, GC, and the mean number of Cs were counted and defined as number of micro-haemorrhages (NEMO), GC and Cs scores, respectively. The European Scleroderma Study Group (ESSG) index constituted the gold standard for DA assessment, and scores ≥3.5 and =3 were considered indicative of high and moderate activity, respectively.

**Results:**

NEMO and GC scores were positively correlated with ESSG index (R = 0.65, *P* <0.0001, and R = 0.47, *P* <0.0001, respectively), whilst Cs score showed a negative correlation with that DA index (R = −0.30, *P* <0.001). The area under the curve (AUC) of receiver operating characteristic plots, obtained by NEMO score sensitivity and specificity values in classifying patients with ESSG index ≥3.5, was significantly higher than the corresponding AUC derived from either GC or Cs scores (*P* <0.03 and *P* <0.0006, respectively). A modified score, defined by the presence of a given number of MHE/MT and GC, had a good performance in classifying active patients (ESSG index ≥3, sensitivity 95.1%, specificity 84.8%, accuracy 88.7%).

**Conclusions:**

MHE/MT and GC appear to be good indicators of DA in SSc, and enhances the role of NVC as an easy technique to identify active patients.

## Introduction

Systemic sclerosis (SSc) is a connective tissue disease clinically characterized by Raynaud’s phenomenon, and progressive fibrotic changes in the skin and internal organs, such as heart, lung, and kidney. Subsets of SSc have been defined, that is, limited cutaneous (lc) SSc, diffuse cutaneous (dc) SSc and SSc without skin involvement [1].

As in other systemic autoimmune diseases, SSc has a clinical course characterized usually by early phases of activity. A small vessel vasculopathy, characterised by peripheral vascular and interstitial mononuclear cell infiltration, represents the hallmark of the disease in the early phase. Conversely, in the late disease stages, the entire process may evolve to irreversible fibrotic changes of the involved tissues and organs [2-4]. This should induce the clinician to define an early diagnosis and recognize any active phase of the disease, in order to contrast the irreversible final damage by introducing an appropriate treatment [5-7].

The assessment of disease activity (DA) in SSc is a rather difficult challenge, and only a composite index that includes several clinical, instrumental, and serological items has, so far, been proposed and validated [8,9].

Nailfold videocapillaroscopy (NVC) is a simple method that allows the clinician to observe and follow up the disease-related micro-vascular changes in an easily accessible capillary bed [10]. NVC plays an important role in the diagnosis of SSc and is included as an item even in the 2013 classification criteria for SSc [11]. In addition, different NVC patterns have been defined and their prognostic value underlined [12-14]. Finally, NVC has been used to define the different phases of the disease. This allows distinguishing patients with early, active, and late NVC pattern, where the active pattern is defined by the presence of numerous ectasic and giant capillaries (GC), ramified capillaries, micro-haemorrhages (MHE) and micro-thrombosis (MT), and initial loss of capillaries with avascular areas [15].

In the present study, we reconsider the specific NVC abnormalities that have been suggested to be increased (MHE, MT, and GC), or progressively reduced (number of capillaries, Cs) during the active phases of disease, to verify whether some quantitative combination of these features could be useful to identify patients with a relevant level of DA.

We decided to assess the presence of MT together with that of MHE separately from that of GC.

This statement was based on the present interpretation of micro-vascular NVC abnormalities in SSc. It has been suggested that MT and MHE are strictly related to each other, and represent the final evolution of enlarged capillary loops in a rapidly progressive pathological process [16]. Conversely, GC formation is certainly the first response to micro-vascular aggression and damage [17], but they may remain substantially unchanged when repeatedly observed in NVC in a slowly evolving or stable disease [18]. Consequently, the progressive reduction of the numbers of Cs can be regarded as the final result of capillary bed pathological aggression, and also of the substantial failure of capillary reconstruction attempts [17,18].

## Methods

### Patients

One hundred and seven patients who met the American College of Rheumatology/European League Against Rheumatism (ACR/EULAR) classification criteria for SSc [11] formed the study population. They were also classified as having lcSSc or dcSSc following the LeRoy criteria [1]. Current pregnancy, diabetes, smoking and onicophagic habitus, presence of anti-phospholipid antibodies, and assumption of beta blockers, all conditions that may influence the NVC pattern, were considered as exclusion criteria from the study. At the moment of study enrolment, 49 out of the 107 patients were treated with infusion of intravenous prostanoids (42 with monthly iloprost and 7 with weekly alprostadil), and 8 were taking bosentan. Furthermore, all of the enrolled patients were under treatment with low-dose acetylsalicylic acid.

### Nailfold videocapillaroscopy

The nailfold capillaries of all fingers of both hands, excluding thumbs, were examined in each patient using a videocapillaroscope with a 200× magnification lens. Four consecutive 1-mm fields for a total extension of 4 mm in the middle of nailfold were examined. The derived digital images were then stored and analysed by using dedicated software (Videocap Scalar Co. Ltd, DS MediGroup, Milan, Italy).

**Figure 1:**
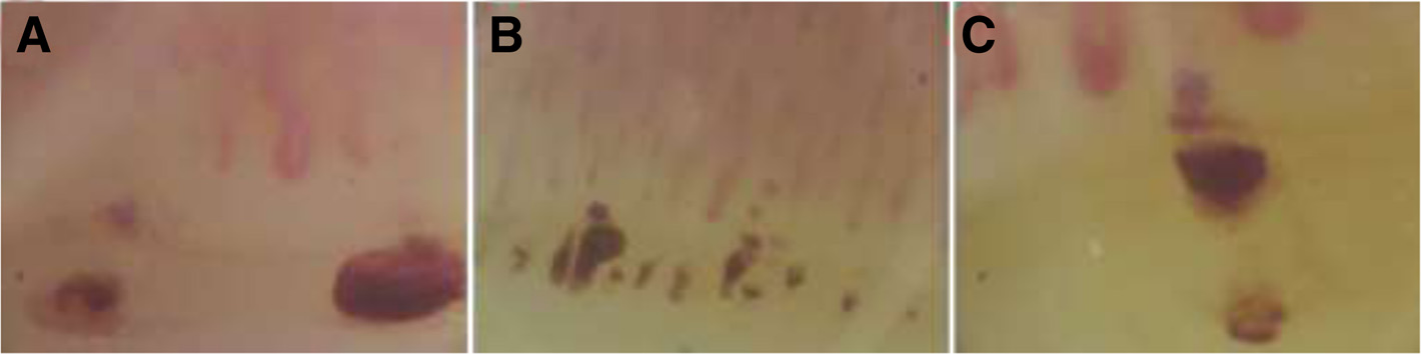
**Example of haemosiderin deposits taken into account when calculating the NEMO score. (A)** Two micro-haemorrhages of relevant size are evident in the cuticle border, as a result of erythrocyte extravasation following the rupture of capillaries. The consequent haemosiderin deposits assume a round form; **(B)** numerous synchronous micro-haemorrhages are observed aligned in the distal row of the cuticle. Synchronicity of events is defined by the presence of haemosiderin deposits in the distal row of capillaries aligned at the same level [16]; **(C)** a haemosiderin deposit that mirrors the shape of a capillary loop is shown. This aspect is considered indicative of a micro-thrombosis [19]. NEMO, number of micro-haemorrhages.

In Figure 1 some typical examples of what we have considered as capillaroscopic MHE and MT are shown [19].

The cumulative number of MHE and MT observed in the images obtained from eight fingers in each patient was calculated, and defined as the NEMO score (number of micro-haemorrhages). Each separate MHE and MT was counted as one in this score, independently of its size. Similarly, the total number of GC, defined as capillaries with a diameter over 50 μm [17], and the mean number of normal or slightly dilated Cs observed in the same NVC fields, were also counted in each patient, and defined as the GC and Cs scores, respectively.

The NVC images were used to subclassify patients in different disease stages, according to a previously proposed method [15] that indicated three different capillaroscopic patterns: (i) ‘early pattern’ , classified as initial stage of disease, characterized by the presence of few GC and capillary MHE, no evident loss of Cs and relatively well-preserved capillary distribution; (ii) ‘active pattern’ , classified as the acclaimed stage of disease, characterized by numerous GC and capillary MHE, moderate capillary loss and mildly disorganized capillary architecture; and (iii) ‘late pattern’ , as advanced disease, with few or absence of GC and MHE, extensive capillary loss, and severe disorganization of the normal capillary architecture.

### Clinical work-up and assessment of disease activity

The European Scleroderma Study Group (ESSG) index was taken as the gold standard for DA assessment [8,9]. This a composite scoring system that includes a number of clinical, instrumental, and serological items, derived by a multivariate analysis performed in a cohort of patients with both lcSSc and dcSSc [8]. A second study carried out in a completely different cohort of patients allowed to finally demonstrate that this scale was a reliable and a valid method to assess DA in SSc [9]. Following the ESSG indications, a score ≥3.5 was considered to have a good sensitivity and specificity in separating patients with high DA level. In addition, we also took into account a cut-off value of 3.0 [20] in order to also include patients with moderate level of DA. A clinical work-up needed for ESSG score definition was completed within the month of NVC performance.

The modified Rodnan skin sScore (mRSS), that is, an important item also included in the ESSG activity index, was performed by experienced physicians (NDP, WM, EZ) other than those who performed NVC (DS, GS). This score is the most widely used method to assess the skin thickening by the exploration of 17 different body sites. The clinician scores skin thickness on a scale ranging from 0 to 3. The mRSS has showed to be a valid method to assess skin involvement in SSc, closely related to the skin histological features, and to have a good inter-observer reliability [21-23].

### Statistical analysis

Statistical analysis was performed by standard procedures using IBM SPSS™ Statistics 21 (IBM, Armonk, NY, USA) and GraphPad Prism™ 6 software package (GraphPad Software Inc., La Jolla, CA, USA).

Since NEMO, GC and Cs scores did not appear to have a normal distribution, we used non-parametric tests to compare these variables with other categorical or ordinal variables taken into account in the study.

A logistic regression model was also tested to assess the contribution of defined values of NEMO, GC and Cs scores in predicting the presence of different level of DA according to predefined ESSG cut-off values.

Receiver operating characteristic (ROC) curves were constructed by plotting sensitivity and specificity values of NEMO, GC and Cs scores in correctly classifying patients having or not having an active disease phase. The Hanley-McNeil test was applied to test the significance of the differences between the areas under the ROC curves obtained from the analysed NVC variables.

Weighted Cohen’s k statistics were preliminarily applied to evaluate the interobserver agreement in both counting NVC abnormalities and assessing mRSS, and resulted to be good in both cases (0.69 and 0.75, respectively).

No correction of the statistical results was made for the presence of missing values, since no data were missed in our database.

### Ethical rules

This study was conducted according to the Helsinki Declaration and approved by the ethics committee of ‘Ospedale G. Pini, Milan, Italy’ , where the study was carried out and all the study patients were recruited. A written informed consent was obtained from all of the enrolled patients.

## Results

The patients with SSc had a mean age of 55.8 yrs. (range 18 to 84 yrs.) and mean disease duration of 6.1 yrs. (range 0 to 30 yrs.). As expected, females represented the large majority of the study population (97/107). The main demographic, clinical and laboratory data of the patients are summarised in Table 1.

**Table 1:** **Demographic, clinical, and serological features, according to ESSG index** [8,9]

	**Whole cohort**	**Patients with lcSSc**	**Patients with dcSSc**
**Patients** (F/M)	107 (97 F, 10 M)	57 (53 F, 4 M)	50 (44 F, 6 M)
**Mean age** (range) (yrs.)^1^	55.8 (18-84)	58.1 (28-84)	53.3 (18-84)
**Mean disease duration** (range) (yrs.)	6.1 (0.5-30)	7.4 (0.5-30)	4.8 (0.5-20)
**ESSG index ≥3.5** (no. of pts.)^2^ (%)	32 (29.9%)	18 (16.8%)	14 (13.1%)
**Mean mRSS** (range) (yrs.)	3.1 (0-32)	2.4 (0-5)	3.5 (0-32)
**Scleredema** (no. of pts.) (%)	60 (56.1%)	32 (56.1%)	28 (56.0%)
Δ^**3**^ **Skin involvement** (no. of pts.) (%)	18 (16.8%)	11 (19.2%)	7 (14.0%)
**Ulcers** (no. of pts.) (%)	22 (21.5%)	11 (19.2%)	11 (22.0%)
Δ^**1**^ **Vascular features** (no. of pts.) (%)	25 (23.4%)	14 (24.5%)	11 (22%)
**Arthritis** (no. of pts.) (%)	5 (4.7%)	3 (5.2%)	2 (4%)
**DLCO <80%** ^**4**^ (no. of pts.) (%)	67 (63.2%)	35 (61.4%)	32 (64.0%)
Δ^**1**^ **Cardiopulmonary features** (no. of pts.) (%)	20 (18.7%)	10 (17.5%)	10 (20.0%)
**ESR >30 mm/h** (no. of pts.) (%)	32 (29.9%)	14 (24.5%)	18 (36.0%)
**Hypocomplementemia** (no. of pts.) (%)	12 (11.7%)	9 (15.7%)	3 (6.0%)

^1^Yrs. = years; ^2^pts = patients; ^3^worsening in the specific organ/system involvement; ^4^a diffusing lung capacity for carbon monoxide below 80% of predicted value. ESSG, European Scleroderma Study Group; lcSSc, limited cutaneous systemic sclerosis; dcSSc, diffuse cutaneous systemic sclerosis; mRSS, modified Rodnan skin score; DLCO, diffusing capacity; ESR, erythrocyte sedimentation rate.

In accordance with the ESSG index pre-defined cut-off value suggested to distinguish patients with high level of DA, that is, an ESSG index ≥3.5, 32 out of the 107 patients could be considered as very active (14 with dcSSc and 18 with lcSSc). Conversely, active patients became 41 when an ESSG index ≥3.0 was used as cut-off value to also included patients with moderate DA (26 with lcSSc and 15 with dcSSc). The main clinical characteristics of nine additional active patients were reported in Table 2. It appears evident that all of these patients showed relevant aspects suggesting a phase of DA.

**Table 2:** **Disease activity assessment of nine patients with ESSG score = 3**

**Patient code**	**15**	**37**	**43**	**53**	**65**	**69**	**89**	**90**	**93**
**mRSS**	0	2	4	5	8	4	6	6	0
**Scleredema**	-	+	+	+	+	+	+	+	+
Δ^**1**^ **Skin features**	-	-	-	-	+	-	-	-	-
**Ulcers**	-	-	+	-	-	-	-	+	+
Δ^**1**^ **Vascular features**	-	-	-	-	+	+	+	-	+
**Arthritis**	+	-	-	-	-	-	+	-	-
**DLCO <80%** ^**2**^	+	+	+	+	-	+	-	+	-
Δ^**1**^ **Cardiopulmonary features**	+	+	-	+	-	-	-	-	-
**ESR >30 mm/h** ^**3**^	+	+	-	+	-	-	-	-	-
**Hypocomplementemia**	-	-	-	-	-	-	-	-	-
**NVC pattern**	Active	Active	Active	Early	Early	Active	Active	Active	Active
**NEMO score**	7	12	3	0	3	6	19	9	39
**GC score**	9	3	7	2	5	3	10	11	5
**Cs score**	7	8	7	9	9	5	8	7	7

^1^Worsening in the specific involvement; ^2^a diffusing lung capacity for carbon monoxide below 80% of predicted value; ^3^an erythrocyte sedimentation rate over 30 mm/h. ESSG, European Scleroderma Study Group; mRSS, modified Rodnan skin score; DLCO, diffusing capacity; ESR, erythrocyte sedimentation rate; NVC, nailfold videocapillaroscopy; NEMO, number of micro-haemorrhages; GC, giant capillaries; Cs, capillaries.

The NEMO score appeared to be a good predictor for DA since NEMO values were strictly correlated with both ESSG index scores (Spearman’s R =0.65, *P* <0.0001), and with mRSS (R =0.59, *P* <0.001), but not with patients’ age and disease duration. When the other components of ESSG activity index were considered, NEMO score appeared to be significantly higher in patients presenting with scleredema (Mann-Whitney *U* Z value =4.59, *P* <0.0001), worsening of skin (Z =5.79, *P* <0.0001), cardio-pulmonary (Z = 3.59, *P* <0.0005), and vascular features (Z =3.18, *P* <0.002), current digital ulcers (Z = 2.91, *P* <0.005), and erythrocyte sedimentation rate (ESR) over 30 mm/h (Z = 3.79, *P* <0.0001).

Similarly, also the GC score was significantly correlated with ESSG index score and with mRSS (R = 0.47, *P* <0.0001, and R = 0.34, *P* <0.001, respectively). Among the different components of ESSG scoring system, GC score was significantly associated with the presence of scleredema (Z = 4.8, *P* <0.0001), digital ulcers (Z = 2.80, *P* <0.01) and worsening of cutaneous (Z = 3.72, *P* <0.0005), vascular (Z = 2.64, *P* <0.01), and cardio-pulmonary (Z = 2.12, *P* <0.05) features.

As expected, negative correlations were found between Cs score and both ESSG index and mRSS (R = −0.27, *P* = 0.004, and R = −0.26, *P* = 0.007). Furthermore, Cs score was significantly lower in patients with scleredema (Z = −3.02, *P* = 0.003), digital ulcers (Z = −3.08, *P* = 0.002), and diffusing capacity (DLCO) <80% of predicted value (Z = −3.4, *P* = 0.001).

**Figure 2:**
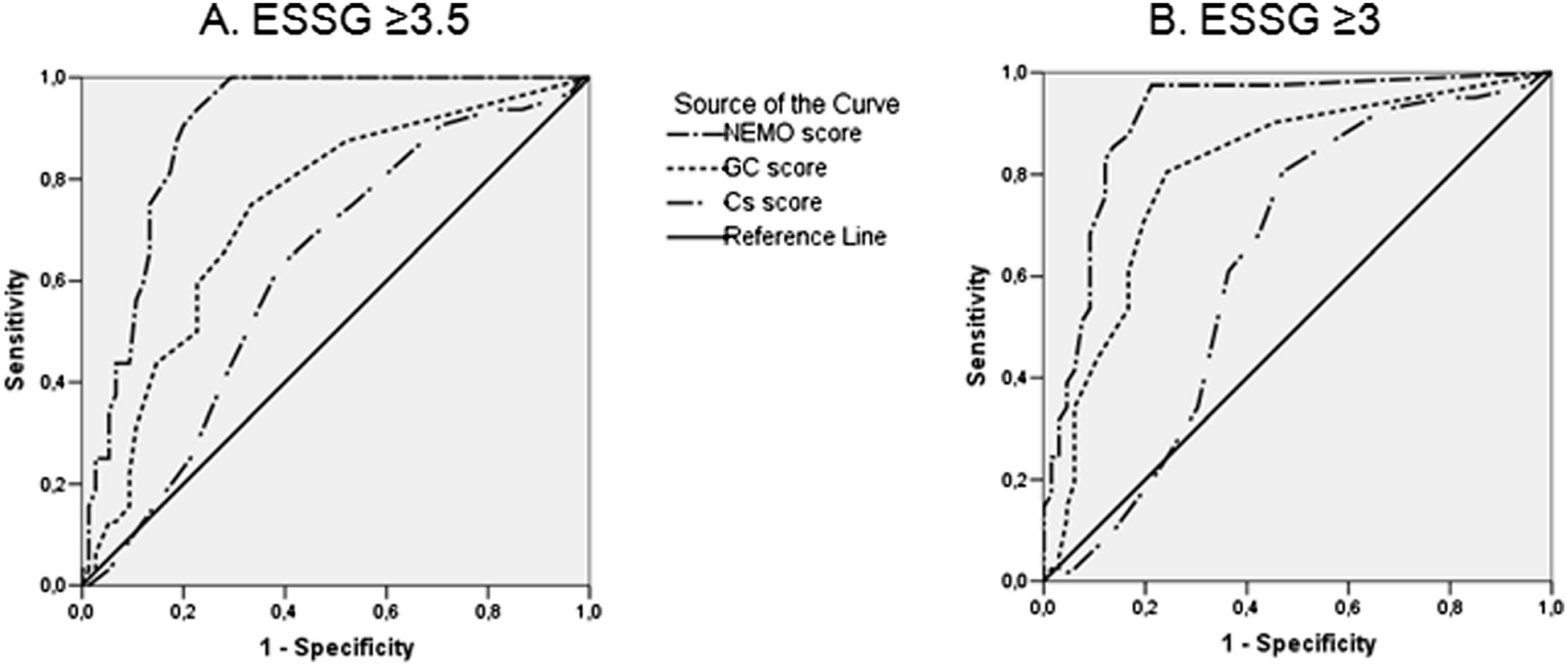
**ROC curves obtained by plotting the sensitivity and 1-specificity values of NEMO, GC and Cs scores in classifying active patients (taking into account either a ESSG index ≥3.5 or ≥3).** Cs ROC curve was plotted using sensitivity and the reciprocal values of specificity considering the maximum value observed as 1 (15, patient number 37). This was done to obtain a plot over the reference line comparable to those obtained for the NEMO and GC ROC plot. When the ROC curves obtained considering a ESSG ≥3.5 as the cut-off value for DA were evaluated (panel **A**), the area under the curve (AUC) defined for the NEMO score (AUC = 0.89, 95% CI 0.84 to 0.95) was significantly greater than that derived from both GC (AUC = 0.73, 95% CI 0.63 to 0.83) and Cs scores (AUC 0.64, 95% CI 0.51 to 0.73). When a ESSG cut-off value ≥3 was considered as indicative of DA (panel **B**), the AUC of the NEMO ROC curve (AUC 0.91, 95% CI 0.84 to 0.96) was significantly higher than that derived from GC score (AUC 0.8, 95% CI 0.71 to 0.83) and Cs score (AUC 0.63, 95% CI 0.52 to 0.74). AUC, area under the curve; CI, confidence interval; Cs, capillaries; DA, disease activity; ESSG, European Scleroderma Study Group; GC, giant capillaries; NEMO, number of micro-haemorrhages; ROC, receiver operating characteristic.

The ROC curves constructed by plotting the sensitivity and specificity values of different NEMO, GC and Cs scores in correctly classifying active patients defined by ESSG index cut-off values of both 3.5 and 3.0 were represented in Figure 2. Generally speaking, NEMO score works better with respect to GC and Cs scores in identifying patients with active disease, and the area under the curve (AUC) of the NEMO score ROC plots were higher than the corresponding AUC of either GC or Cs ROC curves. Both differences reached the level of significance (*P* <0.03 and *P* <0.0006, respectively, considering the two-tailed Hanley-McNeil test) when the ESSG index cut-off value of 3.5 was taken into account. Similarly the AUC of the NEMO score was significantly higher than that of the GC score and Cs score when the ESSG index cut-off value of 3.0 was considered (*P* <0.05 and *P* <0.0001, respectively; see Figure 2 for details).

A NEMO score ≥6, a GC score ≥3, and a Cs score ≤6 showed the most balanced performance in terms of sensitivity/specificity ratio and the best accuracy in correctly classifying patients with active disease at both cut-off values considered (Table 3).

**Table 3:** **Performance of NEMO, GC, and Cs scores in classifying patients with different ESSG scores**

**NEMO score ≥6 for ESSG ≥3**		**NEMO score ≥6 for ESSG ≥3.5**	
Sensitivity	82.9	Sensitivity	87.5
Specificity	87.9	Specificity	81.3
Accuracy	86.0	Accuracy	83.2
**GC score ≥3 for ESSG ≥3**		**GC score ≥3 for ESSG ≥3.5**	
Sensitivity	70.7	Sensitivity	65.6
Specificity	80.3	Specificity	72.0
Accuracy	76.6	Accuracy	70.0
**Cs score ≤6 for ESSG ≥3**		**Cs score ≤6 for ESSG ≥3.5**	
Sensitivity	40	Sensitivity	40.6
Specificity	62.5	Specificity	70.6
Accuracy	54.6	Accuracy	61.1

NEMO, number of micro-haemorrhages; GC, giant capillaries; Cs, capillaries; ESSG, European Scleroderma Study Group.

When the distribution of NEMO score according to the NVC patterns was analysed, it appears evident that the highest NEMO score values and majority of patients with NEMO scores ≥6 were found in the population of patients classified as having the ‘active’ NVC pattern [15] (27/43, 62,8%). However, a relevant number of patients with a NEMO score ≥6 could also be found in the ‘early’ and ‘late’ SSc population [15]. Eight out of eleven patients with the early and all of the five with the late scleroderma pattern having a NEMO score ≥6 had an ESSG index ≥3.5.

When a NEMO score ≥6, a GC score ≥3 and a Cs score ≤6 were analysed all together as predictors of an active phase of disease (ESSG score ≥3.0), by building a logistic regression model, only NEMO and GC variables gave a significant contribution to the model, although the odds ratio of NEMO score was much higher with respect to that of GC score (Table 4).

**Table 4:** **Multiple logistic regression model evaluating the statistical contribution of given values of different NVC scores as a predictor of DA (ESSG ≥3)**

**Variables**	**Odds ratio**	**95% CI** *	***P*** **value**
**NEMO score ≥6**	22.05	6.98-69.64	<0.0001
**GC score ≥3**	3.62	1.14-11.5	0.02
**Cs score ≤6**	2	0.57-7.5	0.26

*95% confidence interval. NVC, nailfold videocapillaroscopy; DA, disease activity; ESSG, European Scleroderma Study Group; NEMO, number of micro-haemorrhages; GC, giant capillaries; Cs, capillaries.

With this figure in mind, we tested sensitivity, specificity and accuracy of a modified (m) NEMO score where a classification of the patients as active was indicated by the presence of ≥6 MHE/MT, or - alternatively - by the contemporary presence of <6 MHE/MT plus a variable number of GC. A mNEMO defined by the presence of ≥6 MHE/MT, or alternatively by the presence of three to five MHE/MT plus at least three GC showed the best accuracy (88.7%) in correctly classifying patients with either moderate or high active disease (with an ESSG score ≥3), with a sensitivity of 95.1% and a specificity of 84.8%., and an odds ratio of 109.2 (95% CI 22.7 to 526.1, *P* <0.0001).

## Discussion

In this study, we demonstrated that the presence of a given number of MHE and MT is highly indicative of an active phase of disease. Even the presence of a sufficient number of GC, and a reduced number of Cs may suggest the presence of DA, although the strength of correlation is higher for the former NVC abnormalities. A score where the presence of a defined number of MHE plus MT, and of a given amount GC were combined - derived by a logistic regression model - showed the best predictive value in selecting patients with a current level of moderate and high DA.

NVC is a simple technique largely adopted in clinical assessment of patients with SSc and other connective tissue diseases [24]. Different applications have been described for NVC in SSc. First of all, NVC has an important diagnostic value in SSc, and a specific pattern has been described as characteristic of the disorder, generically defined as belonging to the scleroderma spectrum [17]. Moreover, the recent ACR-EULAR classification criteria for SSc include NVC among the proposed diagnostic items [11]. A relevant prognostic value has also been ascribed to NVC, and different NVC patterns have been defined to be predictive for a worse outcome [12-14], and for some specific complications of the disorders [25,26]. Finally, NVC has also been used to subclassify patients with SSc according with the phases of disease course, and different NVC patterns described for early, active, and late disease [15]. Conversely, so far, no studies have been carried out specifically aimed at evaluating the potential utility of NVC in stratifying patients according to the level of DA.

The fact that, according to our results, MHE and MT are the NVC abnormalities more strictly indicative of a current DA status is not completely surprising. SSc is usually defined as a disease of the micro-vascular bed of the skin compartment, but potentially compromising the small vessels of internal organs. The endothelial involvement is considered the ‘primum movens’ of the pathological process in SSc, while migration of mononuclear inflammatory cells in the interstitium, activation of myo-fibroblast and fibroblast lineage, with the consequent increased production of extracellular matrix and then fibrosis, could be considered as the following stages [2-4]. A large body of evidence indicates that the typical response to the initial micro-vascular damage and capillary loss in SSc is represented by the compensatory dilatation of remaining capillary loops with formation of GC [17]. In slowly evolving or stable disease, it is not unusual to observe GC unchanged over time. On the contrary, in progressive disorder, the destiny of enlarged capillaries is thrombotic obliteration followed by extravasation. When these phenomena are synchronous in many capillaries, serial extravasations with hemosiderin deposits can be observed in the NVC, aligned distally in the cuticle [16].

The finding that the presence of a given amount of MHE and MT (more strongly than that of GC) is associated with DA phases in SSc seems to confirm that these specific abnormalities could represent the NVC counterpart of a rapidly evolution of micro-vascular involvement in active disease phases. The fact that the correlation is less strong for GC can be ascribed to the well-know finding that some GC may appear stable over time in sequential NVC examinations, and then their presence cannot reflect an active phase of the disease. Conversely, the reduction in the number of Cs may be regarded as the final outcome of pathological process in the capillary bed, once the capillary reconstruction attempts have failed. Of course, in a longitudinal study, where a rapidly progressive reduction of capillary number may be assessed in two or more consecutive NVC examinations, one can certainly consider this finding as expression of an active evolving phase of the disease. This is not the case of the present cross-sectional study in which we demonstrated that the current state of DA is more closely represented by the presence of MH and MT. This statement is certainly reinforced by the observation that MHE and MT, but not GC, have been described in other disorders where the involvement of micro-vascular compartment is an important pathological aspect, although induced by partially different mechanisms [27-30].

Since in a specific logistic model the presence of a certain number GC appear to be also predictive for DA, although to a lesser extent, we checked whether a given combination of all these NVC abnormalities could better identify patients with a relevant DA. The modified score (mNEMO score), in which both MHE/MT and GC were included, demonstrated to be actually more accurate and valid for this purpose.

We are, of course, aware that any instrument devoted to assess DA should be not only valid and reliable but also sensitive to change. Not a cross-sectional study like the present one, but a longitudinal study, where the instrument is used to assess changes of DA over time, is certainly needed to verify this latter characteristic.

The here proposed NVC scoring system named mNEMO appears a feasible instrument to assess DA in SSc, since it is possible to predict an active phase of disease during an outpatient visit and in a rather short time. On the contrary, to evaluate the same entity with the previously proposed methods implies a complicate and time-consuming patient work-up. Of course, patients in whom this specific NVC approach may suggest an active phase of disease should be addressed to a more careful clinical, instrumental and serological evaluation to confirm the suspicion and more precisely define the specific clinical profile. Once a patient has been definitely classified as being in an active phase of disease, a more aggressive therapeutic strategy should be taken into consideration, in accordance with the present recommendations that suggest treating active patients as soon as possible, in order to tentatively modify the natural course of SSc.

## Conclusions

Some specific features observed during NVC examination, such as MHE/MT and GC are strongly predictive of moderate or high level of DA in SSc. A scoring system derived by these findings has been developed and proposed.

## Abbreviations

AUC: area under the curve
CI: confidence interval
Cs: capillaries
DA: disease activity
dc: diffuse cutaneous
DLCO: diffusing capacity
ESR: erythrocyte sedimentation rate
ESSG: European Scleroderma Study Group
GC: giant capillaries
lc: limited cutaneous
MHE: micro-haemorrhages
mNEMO: modified NEMO score
mRSS: modified Rodnan skin score
MT: micro-thrombosis
NEMO: number of micro-haemorrhages
NVC: nailfold videocapillaroscopy
ROC: receiver operating characteristic
SSc: systemic sclerosis
